# Polystyrene nanoplastics induce oxidative stress in *Aurelia coerulea* polyps, microglia, and mice

**DOI:** 10.3389/fimmu.2025.1609208

**Published:** 2025-09-03

**Authors:** Mingshuai Song, Zhenyu Wei, Jingqiang Wang, Liangzhi Li, Xiangyu Li, Xiaofen Ma, Marina Pozzolini, Xinyan Liu, Liang Xiao, Ping Zhong

**Affiliations:** ^1^ School of Health Science and Engineering, University of Shanghai for Science and Technology, Shanghai, China; ^2^ Faculty of Naval Medicine, Naval Medical University, Shanghai, China; ^3^ Department of Neurology, Shidong Hospital, Yangpu District, Shanghai, China; ^4^ Shanghai Jiangong Hospital, Shanghai, China; ^5^ School of Marine Science and Ecology, Shanghai Ocean University, Shanghai, China; ^6^ College of Animal Science, Shanxi Agricultural-University, Jinzhong, Shanxi, China; ^7^ School of Pharmacy, Xinjiang Medical University, Urumqi, Xinjiang, China; ^8^ Department of Earth, Environment and Life Sciences (DISTAV), University of Genova, Genova, Italy; ^9^ Traditional Chinese Medicine Surgery, The First Affiliated Hospital of Naval Medical University, Shanghai, China

**Keywords:** polystyrene nanoparticles, *Aurelia coerulea* polyps, microglia, oxidative stress, MAPK signaling pathway, cognitive impairment

## Abstract

**Introduction:**

This study investigates the oxidative stress responses induced by polystyrene nanoplastics (PS-NPs) across three distinct biological models—*Aurelia coerulea* polyps, BV2 microglial cells, and ICR (Institute of Cancer Research) mice. We aimed to explore the involvement of the mitogen-activated protein kinase (MAPK) signaling pathway as a potential mechanism in invertebrate and cellular systems, while evaluating neurobehavioral outcomes *in vivo*.

**Methods:**

Oxidative stress markers including catalase (CAT), total antioxidant capacity (T-AOC), and malondialdehyde (MDA) were quantified in all the three models. Transcriptomic analysis and RT-qPCR validation targeting the MAPK signaling pathway were performed in *Aurelia coerulea* polyps and BV2 microglial cells. Behavioral assessments, including the open field test and novel object recognition test, were conducted in mice to evaluate anxiety-like behavior and cognitive impairment following PS-NPs exposure.

**Results:**

In polyps, PS-NPs exposure resulted in shortened tentacle length and a dose-dependent decrease in T-AOC and CAT activity, along with an increase in MDA levels, indicating oxidative stress. BV2 microglia exhibited intracellular PS-NP accumulation, increased reactive oxygen species (ROS), upregulated inflammatory cytokines, and elevated apoptosis. Transcriptome analysis revealed significant activation of the MAPK signaling pathway in both polyps and BV2 cells. In mice, PS-NPs caused reduced central zone exploration and lower discrimination index scores, consistent with anxiety-like behavior and cognitive dysfunction. Immunohistochemical staining revealed microglial activation in the hippocampus, exhibiting the neurotoxic effects of PS-NPs.

**Discussion:**

While these models represent distinct organisms and biological contexts, all demonstrated consistent oxidative stress responses upon PS-NPs exposure. Although we do not claim direct equivalency across species, the converging evidence from marine, cellular, and mammalian systems highlights the widespread biological risks posed by nanoplastics. These findings provide a foundation for evaluating environmental and public health threats associated with PS-NPs.

## Graphical Abstract

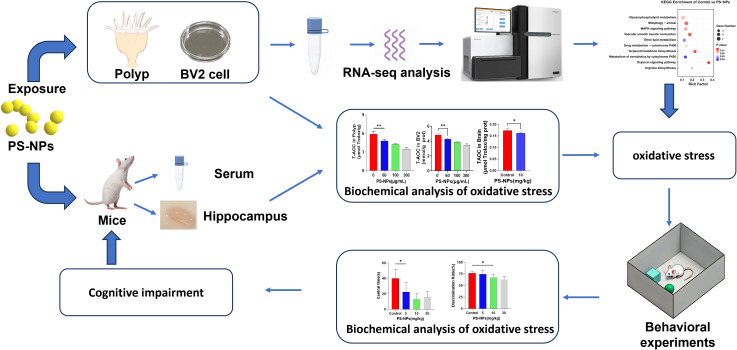

## Highlights

- Exposure to polystyrene nanoplastics (PS-NPs) significantly reduced tentacle length in *Aurelia coerulea* polyps, while increasing catalase (CAT) activity and malondialdehyde (MDA) levels, indicating oxidative stress.
- In BV2 microglial cells, internalized PS-NPs disrupted cellular morphology, increased reactive oxygen species (ROS), and impaired antioxidant capacity.
- Transcriptomic analysis revealed that the mitogen-activated protein kinase (MAPK) signaling pathway is activated in both polyps and BV2 cells.
- Behavioral tests in ICR mice (open field and novel object recognition) showed PS-NPs induced anxiety-like behavior and cognitive impairment.

## 1 Introduction

The exponential rise in plastic production worldwide in recent decades has led to severe environmental contamination, posing substantial threats to ecosystems and human well-being. Every year, millions of tons of plastic waste infiltrate oceans and water bodies, persisting in the environment due to its non-biodegradable nature (1). Microplastics (MPs, 1μm-5mm) and nanoplastics (NPs, typically less than 1μm) are major forms of plastic pollution (2). Due to their smaller size, nanoplastics are more likely to enter organisms, causing cellular damage, autophagy, and inflammation, which can further harm organs (2–4).

Polystyrene (PS), a thermoplastic polymer synthesized via the polymerization of styrene monomers, is widely utilized across various industries due to its excellent molding properties and low-cost characteristics. This large-scale production-consumption model has led to an exponential accumulation of PS waste in the environment. Under environmental weathering processes (e.g., UV radiation, mechanical abrasion), macroscopic PS products gradually degrade into a more hazardous form—polystyrene nanoparticles (PS-NPs). PS-NPs can penetrate organisms through the skin, respiratory system, and digestive tract, accumulating in the food chain and negatively affecting growth, reproduction, nutrition, and various physiological functions (5–8). The ingestion of PS-NPs can cause oxidative harm and provoke inflammatory responses (9, 10). Nanoplastics have been observed to be absorbed by the digestive organs of mice, subsequently entering the liver and swiftly accumulating in adipose tissue (11). Recent findings have identified polystyrene microplastic particles in human feces, colon tissue, lung tissue, placenta, and whole blood (12–16). Nanoparticles, recognized as emerging pollutants, can enter the human circulatory system through inhalation or ingestion. NPs have the ability to cross the blood-brain barrier via olfactory and vascular pathways, influencing the release of neuroinflammatory mediators (e.g., cytokines and chemokines) and altering the expression of transport proteins and receptor markers. In such cases, neurotoxicity and brain dysfunction may occur, posing a significant risk for the development of neurological disorders (17).

The polyp stage of the moon jellyfish (*Aurelia aurita*) displays a simple anatomical structure primarily consisting of the ectoderm and endoderm, with a central gastric cavity encircled by tentacles and a nerve network (18). Polyps are highly sensitive to environmental changes and serve as key bioindicators in marine ecosystems. Their responsiveness makes them an ideal model for studying the accumulation and toxic effects of nanoplastic contamination on marine life (19). Microglia, the intrinsic immune cells of the central nervous system, serve as a crucial model for investigating neuro-oxidative stress due to their role in monitoring and responding to neural damage and pathological states (20). Microglia can undergo functional polarization in response to various stimuli, exhibiting either a pro-inflammatory M1 phenotype or an anti-inflammatory M2 phenotype. M1 markers, such as CD32 and CD11b, are typically associated with enhanced inflammatory responses and increased production of reactive oxygen species (ROS), whereas M2 markers, including IL-10 and Arg-1, are involved in tissue repair and immunosuppression. Under conditions of oxidative stress, microglia play a pivotal role, encompassing the generation and elimination of ROS and the modulation of inflammatory responses (21). Therefore, the utilization of hydroids and BV2 cells to explore the oxidative stress induced by polystyrene nanoplastics holds significant importance.

To systematically investigate the oxidative stress and potential neurotoxic effects induced by PS-NPs, this study integrated three distinct biological models: *Aurelia coerulea* polyps (a marine ecological model), BV2 microglial cells (a neural cell model), and mice (a whole-animal behavioral model), aiming to elucidate the toxicological impacts of PS-NPs from the cellular to the organismal level. This study conducted multiple assessments on *Aurelia coerulea* polyps and BV2 cells to evaluate their antioxidant defense responses to PS-NP-induced oxidative stress. The results showed a significant decline in antioxidant capacity with increasing concentrations of PS-NPs. Transcriptomic analysis confirmed that the oxidative stress triggered by PS-NPs is primarily mediated through the activation of the MAPK signaling pathway. Furthermore, behavioral experiments revealed that the accumulation of PS-NPs in mice induces anxiety-like behaviors and impairs cognitive functions. These findings not only highlight the potential hazards of nanoplastics to marine organisms and the nervous system but also provide a scientific foundation for developing environmental protection and health safety strategies.

## 2 Materials and methods

### 2.1 Experimental materials

Green fluorescent PS-NPs with a diameter of 50 nm (Xi’an Qiyue Biological Company, China) were procured in the current study. *Aurelia aurita* polyps were provided by the Naval Medical University. Institute of Cancer Research (ICR) mice (male, aged between 6 to 8 weeks) were acquired from the Animal Experiment Center of the Naval Medical University. The BV2 microglial cell line was sourced from Shanghai Qise Biological Technology Co., Ltd. (Shanghai, China). All animal experiments were carried out following the guidelines outlined in the National Institutes of Health Guide for the Care and Use of Laboratory Animals.

### 2.2 Exposure experiments

*Aurelia coerulea* polyps were exposed to seawater solutions containing PS-NPs at concentrations of 0, 60, 100, 200, and 300 μg/mL for 48 hours. The contraction and extension of the polyps’ tentacles were monitored at 1, 6, 12, 24, and 48-hour intervals using a fluorescence microscope (Excelitas Technologies, USA) to track PS-NP uptake. BV2 cells were seeded in 6-well plates and cultured in DMEM with 10% serum. After reaching 80% confluence, the cells were exposed to PS-NPs at concentrations of 60, 100, 150, and 300 μg/mL for 12 hours, and PS-NP accumulation was observed using fluorescence microscopy. ICR mice were randomly assigned to one control group and three experimental groups (n=13/group). It has been reported that each person consumes approximately 5g of microplastics per week (22). Based on an average adult weight of 60 kg, we designed the exposure dose for mice accordingly. The experimental groups were orally gavaged daily with PS-NPs at 5, 10, and 30 mg/kg, while the control group received PBS. Body weight was monitored throughout the 14-day experiment. Upon completion, blood and brain samples were collected and stored at -80°C for analysis.

### 2.3 Biochemical assays

Total antioxidant capacity (T-AOC) reflects the overall antioxidant capacity of biological samples, catalase activity (CAT) is an important antioxidant enzyme that decomposes hydrogen peroxide, and malondialdehyde (MDA) is a marker of lipid peroxidation indicating oxidative damage. Together, these indicators provide a comprehensive assessment of oxidative stress status, and were measured using commercial kits (Beyotime Biotechnology, China) for each of the three models (polyps, BV2 cells, and mice).

Polyps were exposed to PS-NPs at concentrations of 60, 100, 150, 200, and 300 μg/mL, along with a control group treated with normal seawater. After 48 hours of exposure, T-AOC, CAT, and MDA levels in the hydroids were quantified using commercial kits (n=6). Also, BV2 cells were seeded in six-well plates and cultured to 80% confluence. The medium was then replaced with serum-free medium containing PS-NPs at concentrations of 0, 30, 60, 100, 150, and 300 μg/mL. After 12 hours of exposure, T-AOC, CAT, and MDA levels were measured using commercial kits (n = 6). Finally, mice in the 10 mg/kg group and the control group were anesthetized, and brain tissues were collected through orbital blood sampling and dissection. Serum and hippocampal tissues were then isolated, and their T-AOC, CAT, and MDA levels were measured using commercial kits (n=6).

### 2.4 BV2 cell viability assessment

BV2 cells, cultured in complete medium with 10% FBS, were transferred to 96-well plates at a volume of 100 μL per well, with 4 replicates. The cells were allowed to adhere for 6 hours. Subsequently, the medium was substituted with fresh medium containing PS-NPs at concentrations of 0, 10, 30, 60, 100, 150, 300, and 600 μg/mL (100 μL per well). Following a 12-hour incubation period, cell viability was evaluated using the CCK-8 assay.

### 2.5 Detection of reactive oxygen species

When BV2 cells cultured in six-well plates achieved 80% confluence, the medium was substituted with DMEM containing PS-NPs at concentrations of 0, 60, 100, 150, and 300 μg/mL. Following a 12-hour incubation, reactive oxygen species (ROS) production was quantified using a ROS detection kit (Beyotime, China) according to the manufacturer’s instructions. Flow cytometry (Beckman Coulter, USA) was utilized to evaluate ROS levels in the samples.

### 2.6 Apoptosis assays

BV2 cells were cultured to 80% confluence and exposed to PS-NPs at concentrations of 100, 150, and 300 μg/mL for 12 hours. Apoptosis was assessed using the Annexin V-FITC/PI apoptosis detection kit (Yisheng, China), and flow cytometry was used to analyze the fluorescence signals of Annexin V-FITC and propidium iodide (PI).

### 2.7 Detection of inflammatory factors

BV2 cells at 80% confluence were treated with PS-NPs at concentrations of 30, 60, 100, 150, and 300 μg/mL for 12 hours. RNA was extracted, reverse transcribed into cDNA, and the expression of IL-1β and TNF-α was quantified by SYBR Green real-time PCR. The qPCR protocol was performed in two steps: initial denaturation at 95°C for 30 sec, followed by 40 cycles of denaturation at 95°C for 5 sec and combined annealing/extension at 60°C for 30 sec. Melting curve analysis was conducted from 65°C to 95°C with a ramp rate of 0.5°C/sec. The same qPCR protocol was used throughout this study.

### 2.8 Behavioral experiments

The open field apparatus (50 cm x 50 cm x 40 cm) was divided into two zones: the central area (30 cm x 30 cm) and the peripheral area. Mice were allowed to acclimate to the experimental room for 1 hour before the trial. Each mouse was placed in the central zone and allowed to explore the apparatus for 5 minutes. Key parameters, including total distance traveled, entries into the central zone, and time spent in the central zone, were recorded (n = 5). The apparatus was cleaned with 75% ethanol after each trial to ensure consistency. The Novel Object Recognition (NOR) test consisted of three phases: habituation, training, and testing. During habituation, mice explored an empty box for 10 minutes. In the training phase, two identical objects were placed 10 cm from the box walls, and mice were allowed 10 minutes to explore. The objects used in the test were wooden, non-edible items with distinct shapes—a cone and a cube—designed to eliminate any potential influence from odor or food-related cues. After 24 hours, one object was replaced with a novel one, and mice were given 5 minutes to explore. Activity was recorded via video camera (n = 5). The apparatus was sanitized with 75% ethanol between trials.

### 2.9 IBA-1 immunohistochemical staining

To assess microglial activation in brain tissue, immunohistochemical staining for IBA-1 (ionized calcium-binding adaptor molecule 1) was performed. IBA-1 is a well-recognized marker of microglial activation, and its upregulation reflects morphological and functional changes associated with neuroinflammation. Three animals were selected from both the 10 mg/kg treatment group and the control group. Mice were anesthetized and transcardially perfused with 4% paraformaldehyde (PFA), followed by an additional 12–24 hours of fixation in PFA. Tissues were dehydrated, paraffin-embedded, and sectioned into 5–10 μm slices. Sections were deparaffinized, rehydrated, and subjected to antigen retrieval in heated citrate buffer (pH 6.0). Endogenous peroxidase activity was quenched by incubating sections with 3% hydrogen peroxide for 10 minutes at room temperature. Non-specific binding was blocked using 5% goat serum for 30 minutes at room temperature. The sections were then incubated overnight at 4 °C with a rabbit polyclonal anti-IBA-1 antibody (1:500, Servicebio, China). After PBS washes, sections were incubated with a biotinylated goat anti-rabbit IgG secondary antibody (1:200, Servicebio) for 1 hour at room temperature, followed by the application of the ABC comple. Visualization was achieved using DAB (3,3′-diaminobenzidine) until optimal color development, after which the reaction was stopped by rinsing in distilled water. Sections were dehydrated through a graded ethanol series, cleared in xylene, and mounted with neutral balsam. The distribution and morphology of IBA-1-positive microglia were examined under a light microscope.

### 2.10 Transcriptomic analysis

Total RNA was extracted from both *Aurelia coerulea* polyps and BV2 cells utilizing the Total RNA Extraction Kit (Fyjnbio, China). The RNA quantity and purity were assessed with the NanoDrop ND-1000 (NanoDrop, USA), while RNA integrity was confirmed using the Bioanalyzer 2100 (Agilent, USA) and agarose gel electrophoresis. Samples meeting the criteria of a concentration >50 ng/μL, RIN value >7.0, OD260/280 >1.8, and total RNA >1 μg were deemed suitable for subsequent experiments. Poly (A) RNA was enriched from the total RNA employing Dynabeads Oligo (dT) (Thermo Fisher, USA) and then fragmented at 94°C for 5–7 minutes using the Magnesium RNA Fragmentation Module (New England Biolabs, USA). The fragmented RNA was subsequently reverse-transcribed into cDNA utilizing SuperScript™ II Reverse Transcriptase (Invitrogen, USA).

Following this, the RNA-DNA hybrids were converted into double-stranded DNA using E. coli DNA polymerase I and RNase H (New England Biolabs), with incorporation of dUTP Solution (Thermo Fisher) during end repair. After adding an A base to the 3’ ends, size selection and purification were carried out using magnetic beads. The library underwent treatment with UDG enzyme (New England Biolabs), followed by PCR amplification under the specified conditions, resulting in a library with an average size of 300 bp ± 50 bp. Subsequently, paired-end sequencing (150 bp reads, PE150) was performed on the Illumina NovaSeq™ 6000 system (Illumina, USA) following the standard protocol. The raw sequencing data in fastq format underwent quality control using fastp, which involved removing adapters, duplicates, and low-quality sequences with default parameters. Clean reads were aligned to the Homo sapiens genome (GRCh38) using HISAT2, generating bam format files. Transcript assembly and gene expression quantification in FPKM were conducted using StringTie. Differential gene expression analysis between samples was carried out using the R package edgeR, defining differentially expressed genes as those with a fold change >2 or <0.5 and a p-value <0.05. Gene Ontology (GO) and KEGG pathway enrichment analyses were performed using DAVID.

### 2.11 Validation of MAPK pathway genes

Transcriptomic analysis revealed the critical involvement of the MAPK signaling pathway in PS-NP-induced oxidative stress. To validate this finding, key differentially expressed genes were selected for real-time quantitative PCR (qPCR) analysis. RNA extracted from the identical samples was employed for complementary DNA (cDNA) synthesis. Specific primers tailored for the selected genes were designed, with SYBR Green utilized in the qPCR process to measure gene expression levels accurately. Statistical analysis was performed to contrast the PS-NP treatment groups with the control group, enabling a comprehensive understanding of the impact of PS-NPs on gene expression within the MAPK signaling pathway.

### 2.12 Statistical methods

The quantitative data were presented as mean ± standard deviation (± s). Statistical analyses were performed using SPSS version 26.0 (IBM, USA), employing one-way ANOVA and independent sample t-tests, with the significance level set at α = 0.05. A p-value < 0.05 was considered statistically significant. Graphs were generated using GraphPad Prism version 9.5.1 (GraphPad Software, USA).

## 3 Results

### 3.1 The effects of PS-NPs on *Aurelia coerulea* polyps

**Figure 1:**
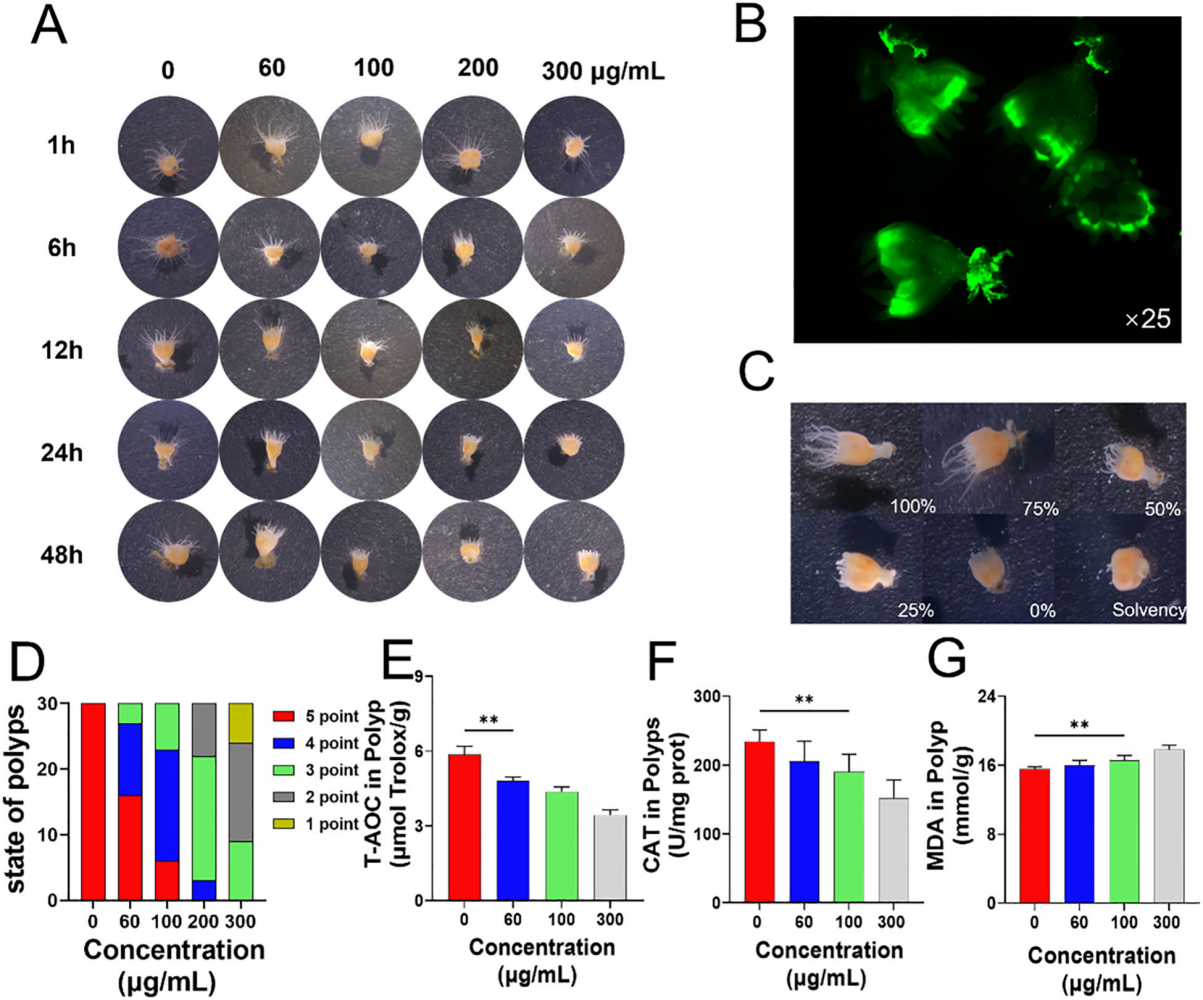
Effects of PS-NPs on *Aurelia coerulea* polyps. **(A)** Morphological changes of *Aurelia coerulea* polyps at different concentrations; **(B)** Distribution of fluorescent PS-NPs in polyps; **(C)** Diagram of the degree of tentacle extension in polyps, 100%: fully extended, 75%: mostly extended, 50%: partially extended, 25%: partially retracted, 0%: fully retracted, Solvency: death; **(D)** Statistical chart of tentacle extension status after 48 hours of exposure to PS-NPs, scoring criteria: 0 points (death), 1 point (<25%), 2 points (25%-50%), 3 points (50%-75%), 4 points (75%-95%), 5 points (95%-100%), 30 individuals per group; **(E–G)** Total antioxidant capacity, catalase activity, and lipid oxidation changes in polyps ( $\bar{\text{x}}$ ± s, n=6). **P < 0.01 indicate significant differences compared to the control group.

*Aurelia coerulea* polyps were cultured in artificial seawater containing varying concentrations of PS-NPs, and their morphology was monitored over a 48-hour period. A notable reduction in tentacle length was observed with escalating PS-NPs concentrations and prolonged exposure time (**Figure 1A**). Following a 48-hour treatment with green fluorescent PS-NPs (100 μg/mL), fluorescence microscopy unveiled the accumulation of PS-NPs in the gastrovascular cavity, around the mouth, and at the basal disc of the polyps (**Figure 1B**). The tentacle extension status of polyps subjected to different PS-NPs concentrations for 48 hours was quantitatively evaluated based on the extension states illustrated in **Figure 1C** (**Figure 1D**). Relative to the untreated controls, the treated polyps exhibited an ellipsoidal morphology. The low-dose group (60-100 μg/mL) displayed sensitivity to the needle reaction, the medium-dose group (200 μg/mL) also exhibited needle reactions, while the high-dose group (300 μg/mL) showed no significant needle reaction. As the PS-NPs concentration increased, the tentacle extension length progressively decreased, aligning with the sensitivity trend in needle reactions. CAT, MDA, and T-AOC can collectively reflect the intracellular oxidative stress status and have high experimental operability and comparability. In polyps, T-AOC decreased significantly with increasing concentrations of PS-NPs, suggesting a compromised antioxidant defense under prolonged oxidative stress (**Figure 1E**). CAT activity declined, while MDA levels increased, indicating elevated oxidative stress (**Figures 1F, G**).

### 3.2 Effects of PS-NPs on BV2 cells

**Figure 2:**
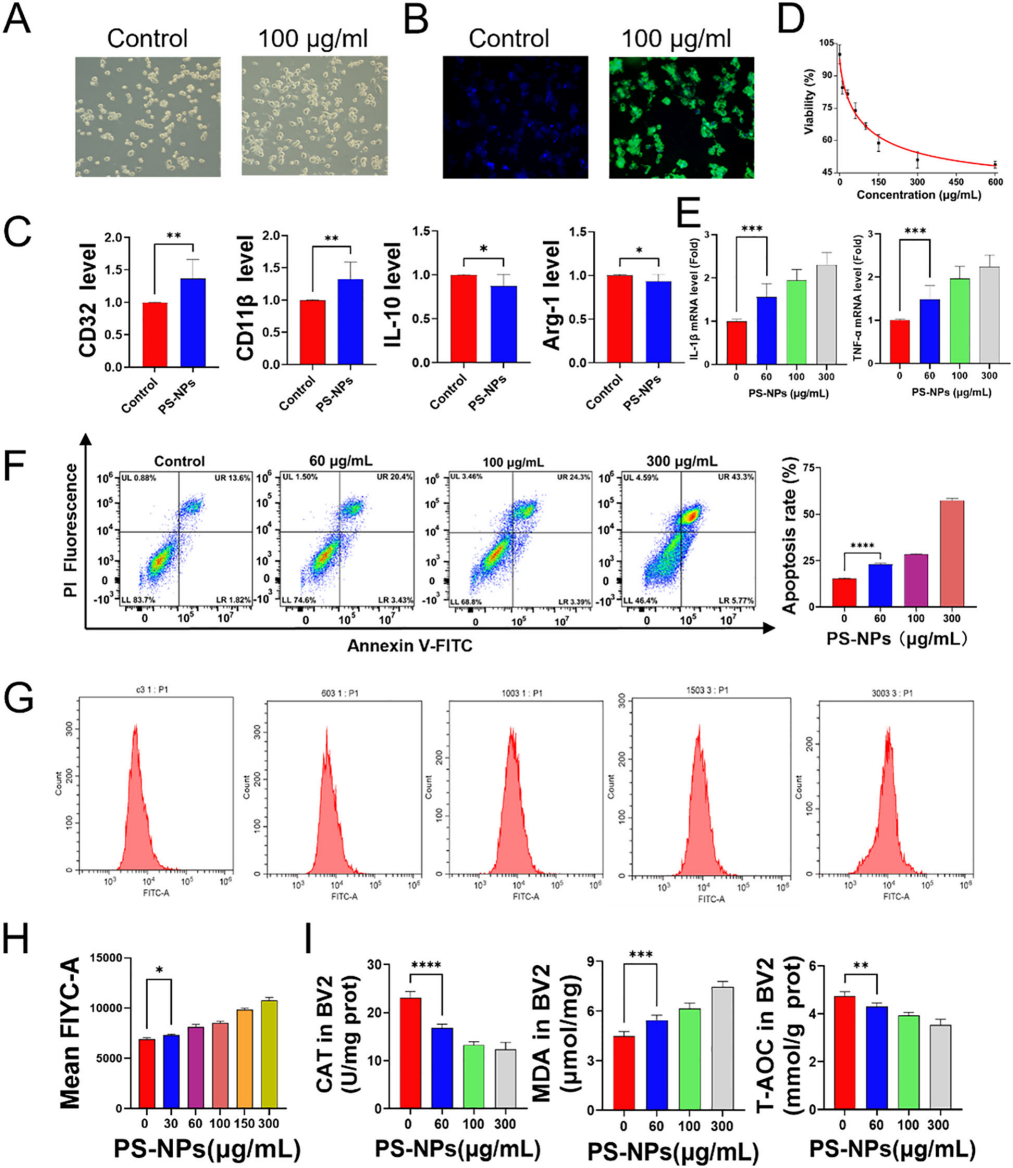
Effects of PS-NPs on BV2 cells. **(A)** Morphological changes in BV2 cells after PS-NPs treatment; **(B)** Phagocytosis of PS-NPs by BV2 cells; **(C)** Gene expression of polarization markers in BV2 cells; **(D)** Changes in cell viability of BV2 cells after treatment with different concentrations of PS-NPs; **(E)** Gene expression levels of pro-inflammatory factors in BV2 cells treated with different concentrations of PS-NPs; **(F)** Apoptosis in BV2 cells after PS-NPs treatment; **(G, H)** PS-NPs induced ROS production in BV2 cells; **(I)** Changes in antioxidant capacity, catalase, and lipid peroxidation in BV2 cells after PS-NPs treatment (n=6). *P < 0.05, **P < 0.01, ***P < 0.001, and ****P < 0.0001 indicate significant differences compared to the control group.

BV2 cells were exposed to culture media containing various concentrations of PS-NPs (ranging from 0 to 600 μg/mL) to evaluate cytotoxicity using the CCK-8 assay. The results indicate a significant decrease in cell viability with increasing PS-NP concentrations. The higher concentration PS-NP treatment groups exhibited a marked reduction in cell viability compared to the control group. This suggests that the toxicity of PS-NPs to BV2 cells is concentration-dependent, with higher concentrations significantly inhibiting normal cellular metabolic functions. Subsequent analyses were conducted using PS-NPs at concentrations of 30, 60, 100, and 300 μg/mL based on the viability outcomes (**Figure 2D**). Immunofluorescence analysis was carried out on BV2 cells treated with 100 μg/mL fluorescently labeled PS-NPs DMEM for 12 hours. The experimental results indicate that, compared to the untreated control group, BV2 cells in the treatment group exhibited a pro-inflammatory phenotype with increased small cell bodies and elongated branches, along with visible intracellular accumulation of PS-NPs marked by green fluorescence (**Figures 2A, B**). Further assessment of cell morphology under bright-field microscopy and PCR analysis targeting M1 polarization markers (CD32, CD11b) and M2 polarization markers (IL-10, Arg-1) demonstrated significant differences between the control group and the 100 μg/mL PS-NPs-treated BV2 cells. Compared to the control group, PS-NPs treatment led to enlarged BV2 microglia with increased protrusions. Moreover, the upregulation of CD32 and CD11b genes in the 100 μg/mL group indicated a shift toward M1 polarization (**Figure 2C**). Gene expression profiling of IL-1β and TNF-α in BV2 cells subjected to PS-NPs for 12 hours revealed heightened levels of both genes in the treatment group compared to the control group (**Figure 2E**), implying that PS-NPs trigger an inflammatory response in BV2 cells. Notably, apoptosis in BV2 cells significantly increased following 12-hour PS-NPs exposure (**Figure 2F**), suggesting that PS-NPs instigate cell apoptosis. The generation of ROS in cells was detected using a flow cytometer. As the concentration of PS-NPs increased, the fluorescence intensity also increased, indicating an elevated production of ROS (**Figures 2G, H**). This indicates that PS-NPs amplify ROS generation in BV2 cells. Upon treating BV2 cells with varying PS-NPs concentrations (0, 30, 60, 100, 150, 300 μg/mL) in serum-free DMEM for 12 hours, CAT and T-AOC levels diminished while MDA levels surged with higher PS-NPs concentrations (**Figure 2I**).

### 3.3 Transcriptome analysis of *Aurelia coerulea* polyps

**Figure 3:**
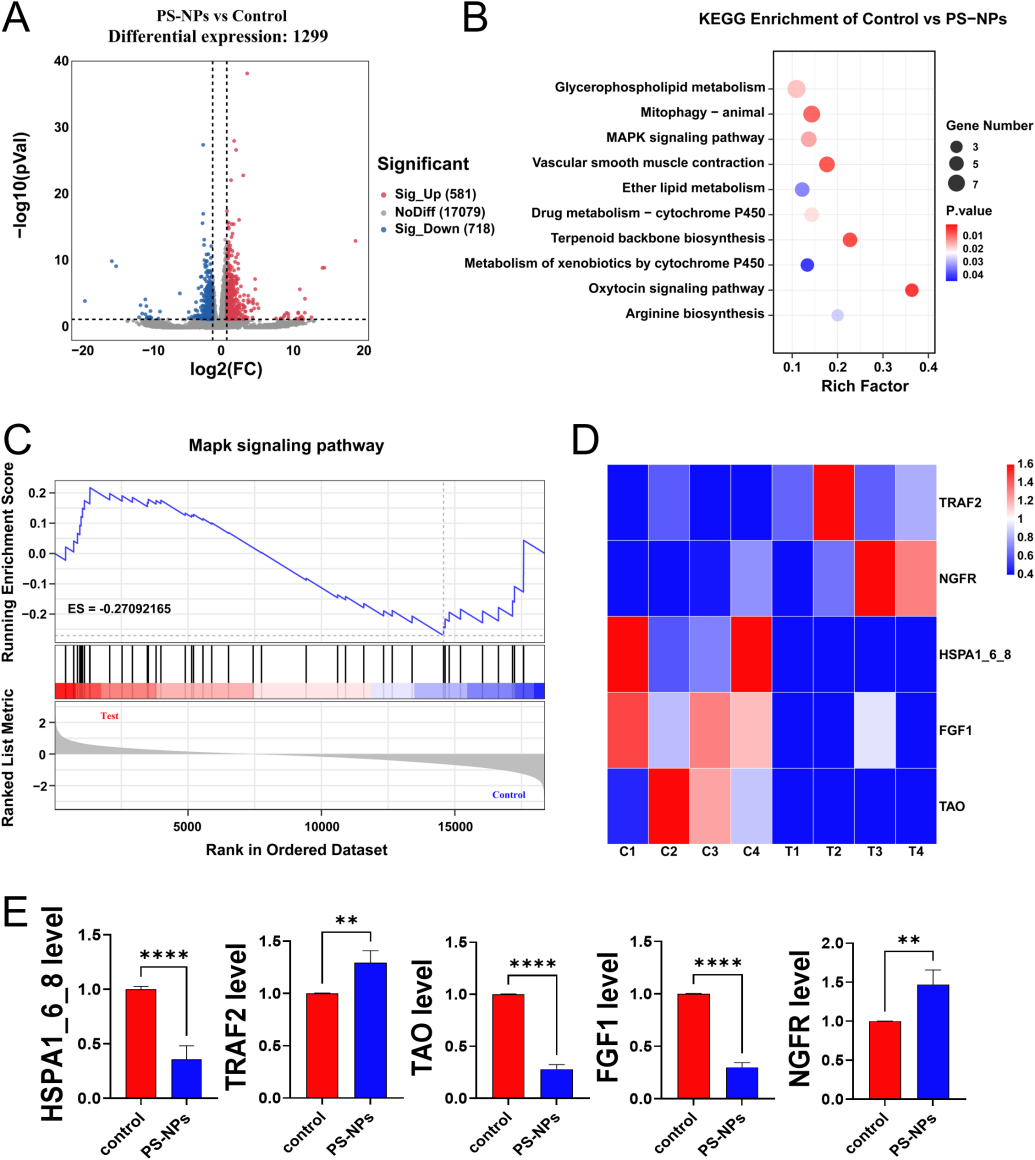
Transcriptome analysis of *Aurelia coerulea* polyps after PS-NPs treatment. **(A)** Volcano plot of differentially expressed genes between PS-NPs treatment group and control group; **(B)** Bubble chart of KEGG enrichment analysis results; **(C)** GSEA plot of the MAPK pathway; **(D)** Heatmap of differentially expressed genes in the MAPK pathway; **(E)** RT-qPCR validation results of MAPK-related genes ( $\bar{\text{x}}$ ± s, n=6). **P < 0.01, and ****P < 0.0001 indicate significant differences compared to the control groups.

The transcriptome analysis of *Aurelia coerulea* polyps exposed to PS-NPs unveiled 1,299 genes exhibiting statistically significant variances, as depicted in the volcano plot (**Figure 3A**). Subsequently, an enrichment analysis of these differentially expressed genes was conducted, with the top ten pathways, determined by the number of differentially expressed genes, visualized in a bubble chart (**Figure 3B**). Gene Set Enrichment Analysis (GSEA) focusing on the MAPK pathway indicated its activation, as showcased in **Figure 3C**. This suggests that exposure to PS-NPs could trigger the activation of the MAPK signaling pathway, potentially leading to oxidative stress in polyps. Notably, within this pathway, five genes exhibited significant differential expression (**Figure 3D**), and their expression levels, validated through RT-qPCR, aligned with the transcriptome data, as demonstrated in **Figure 3E**.

### 3.4 Transcriptome analysis of BV2 cells

**Figure 4:**
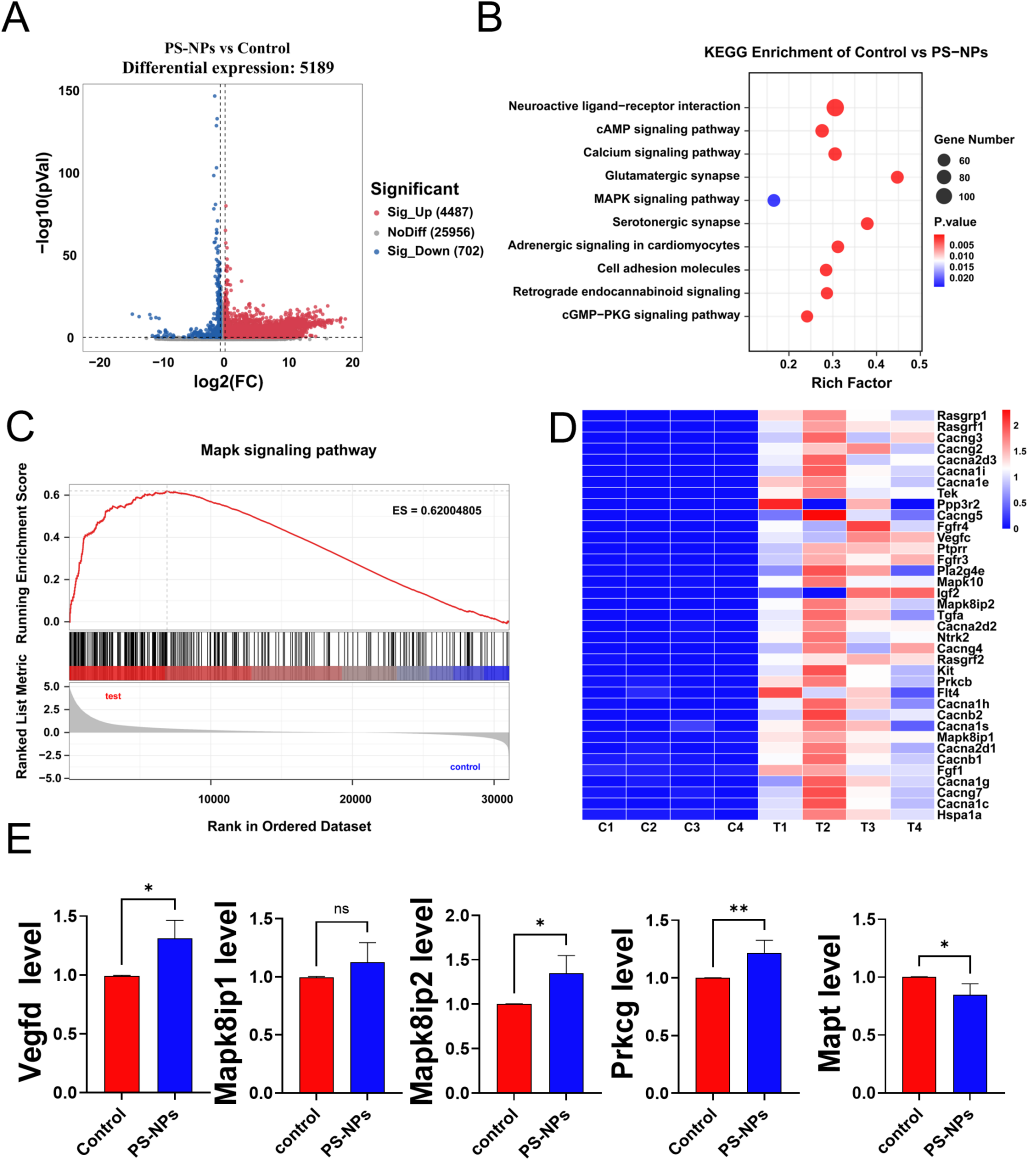
Transcriptome analysis of BV2 cells after PS-NPs treatment. **(A)** Volcano plot of differentially expressed genes between PS-NPs treatment group and control group; **(B)** Bubble chart of KEGG enrichment analysis results; **(C)** GSEA plot of the MAPK pathway; **(D)** Heatmap of differentially expressed genes in the MAPK pathway; **(E)** RT-qPCR validation results of MAPK-related genes ( $\bar{\text{x}}$ ± s, n=8). *P < 0.05 and **P < 0.01 indicate significant differences compared to the control group. “ns” stands for “not significant”.

The transcriptome analysis unveiled 5,189 genes with statistically significant differences, as illustrated in the volcano plot (**Figure 4A**). Subsequent KEGG enrichment analysis was conducted to identify the KEGG pathways associated with these differentially expressed genes. The analysis identified 89 KEGG pathways with significant variances, with the top 10 pathways, based on gene numbers, presented in a bubble chart (**Figure 4B**). Similarly, akin to the transcriptome findings in polyps, differentially expressed genes in BV2 cells post PS-NPs exposure were enriched in the MAPK pathway. Gene Set Enrichment Analysis (GSEA) of the MAPK pathway indicated an overall upregulation of the MAPK signaling pathway (**Figure 4C**), suggesting a potential association of the MAPK signaling pathway with oxidative stress in both BV2 cells and *Aurelia coerulea* polyps. A heatmap was generated to visualize the differentially expressed genes enriched in the MAPK pathway (**Figure 4D**). Five genes with notable FPKM values were selected for RT-qPCR validation, and the outcomes were consistent with the transcriptome data (**Figure 4E**).

### 3.5 Effects of PS-NPs on mice

**Figure 5:**
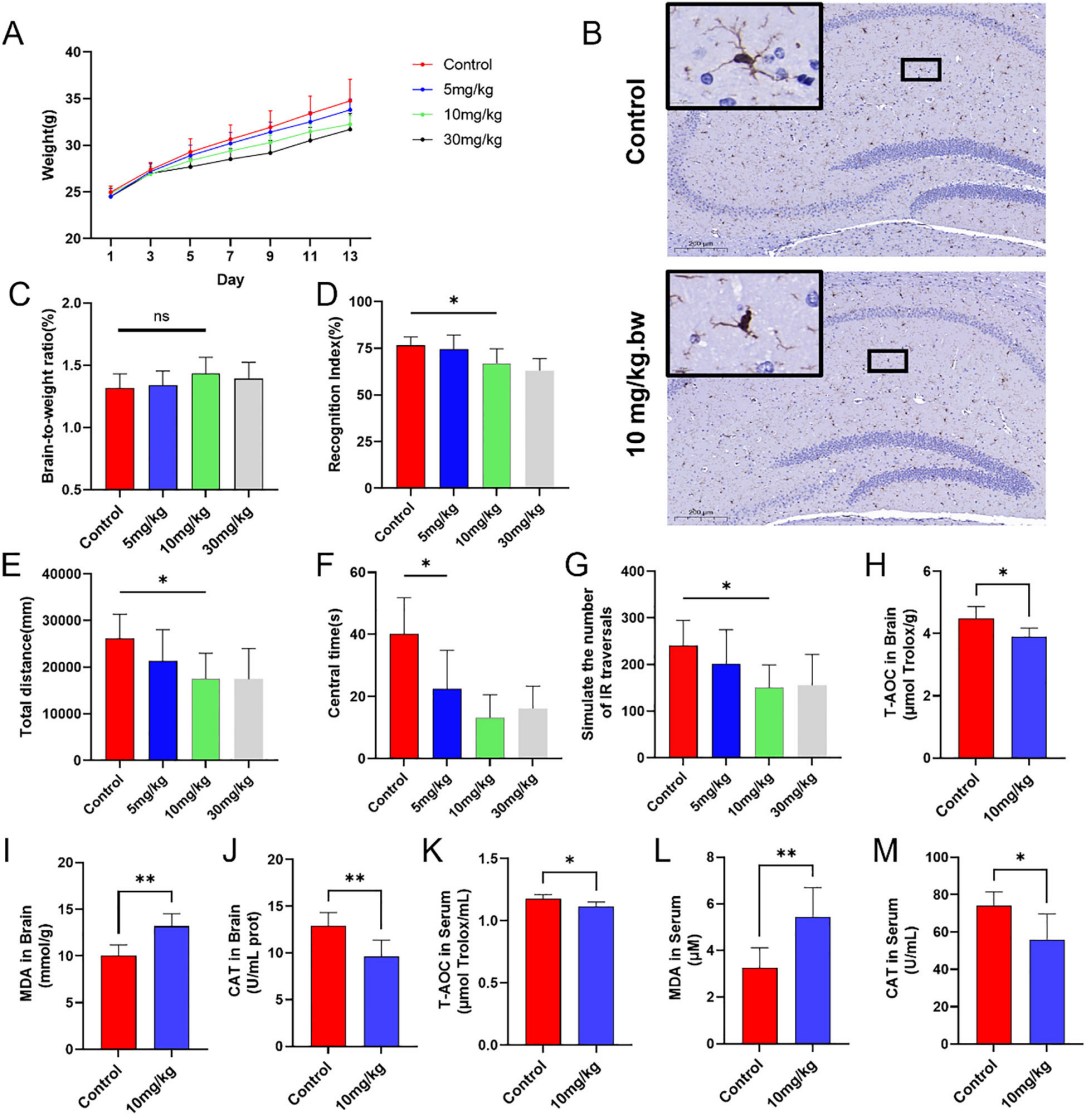
*In vivo* experiments on mice. **(A)** Changes in body weight of mice; **(B)** Representative IHC images of microglia in the hippocampus; **(C)** Brain-to-body weight ratio on day 14; **(D)** Discrimination index of mice in the novel object recognition test across groups; **(E)** Total movement distance of mice in the open-field test; **(F)** Activity time in the central area during the open-field test; **(G)** Simulated infrared crossing counts in the open-field test; **(H-M)** Biochemical analysis of oxidative stress markers in brain tissue and serum. *P < 0.05 and **P < 0.01 indicate significant differences compared to the control group. “ns” stands for “not significant”.

During the PS-NPs exposure feeding period, body weight changes in mice were continuously monitored and recorded. Compared with the control group, the low-concentration exposure group (5 mg/kg) exhibited growth retardation on day 9, while the medium- (10 mg/kg) and high-concentration (30 mg/kg) exposure groups showed similar trends as early as day 3. By day 13, the body weights of mice in all treatment groups were lower than those in the control group (**Figure 5A**). IBA-1 immunohistochemistry demonstrated that oral exposure to PS-NPs activated microglia in the brain. In the control group, hippocampal microglia exhibited a typical ramified morphology with fine, highly branched processes. In the treated group (10 mg/kg), microglial processes appeared noticeably thicker and less branched, indicating morphological activation, while the cell body size remained largely unchanged (**Figure 5B**). Dissection revealed random changes in brain-to-body weight ratios with increasing treatment concentrations (**Figure 5C**).

In the novel object recognition test, all mice underwent three training sessions. Although the discrimination index in the experimental groups decreased to varying degrees, it remained above 0.5. Compared to the control group, the decreases in the discrimination index in the medium-dose group (10 mg/kg) and high-dose group (30 mg/kg) were statistically significant (**Figure 5D**). Mouse activity in the open field test reflected their anxiety levels. Given the central area of the open field was more exposed, mice perceived it and avoided entering it. The experimental groups had significantly lower total movement distance, fewer entries into the central area, and less time spent in the central area, compared to the control group (**Figures 5E–G**). Brain tissue and serum samples were collected from each group of mice to assess potential oxidative stress induced by PS-NPs. Compared to the control group, PS-NPs exposure led to a decrease in CAT levels, an increase in MDA levels, and a reduction in T-AOC in mice (**Figures 5H–M**).

## 4 Discussion

Plastic pollution not only affects individual organisms but also exerts broader impacts on marine ecosystems (23–25). Previous studies have demonstrated that nanoplastics can affect the survival, behavior, and reproduction of fish and aquatic invertebrates, disrupt normal physiological processes, and exacerbate oxidative stress responses (26). In addition, *C. elegans* acs-22 mutants exhibited oxidative stress following exposure to PS-NPs (27). Our results indicate that with increasing PS-NPs exposure, both tentacle extension and stinging responses declined, suggesting that the normal physiological functions of *Aurelia coerulea* polyps may be impaired due to mechanical damage or chemical toxicity caused by PS-NPs.

Although previous studies have explored the oxidative stress responses triggered by nanoparticles in different models, the uniqueness of our study lies in the use of the *Aurelia coerulea polyp* and BV2 cell models to investigate the oxidative stress mechanisms induced by PS-NPs in these models. BV2 microglial cells are a widely used model for studying neuroinflammation and oxidative stress, making them suitable for evaluating the neurotoxic mechanisms of nanoplastics in the central nervous system. *Aurelia coerulea* polyps, as ecologically sensitive marine organisms, serve as effective indicators for assessing the bioaccumulation and toxic effects of nanoplastics in aquatic environments. The combination of these two models allows for a comprehensive investigation of PS-NP-induced neurotoxicity from both cellular and ecological perspectives. Our research demonstrates that PS-NPs induce oxidative stress, increase ROS production, reduce antioxidant enzyme activity, and promote cell apoptosis, which may further exacerbate oxidative damage in the nervous system. Subsequent behavioral tests further confirmed the cognitive impairments in mice caused by PS-NPs, providing more detailed neurotoxicological evidence compared to previous studies.

Oxidative stress in microglia is closely associated with cognitive impairment through the induction of synaptic damage, neuroinflammation, and mitochondrial dysfunction (28). Our study found that PS-NPs can accumulate in BV2 cells and have the ability to penetrate the cell membrane. The phagocytic capability of microglia may further facilitate the internalization of PS-NPs, making organelles, particularly mitochondria, more susceptible to their effects, thereby inducing oxidative stress (29, 30). Relevant studies have found that PS-NPs reduce mitochondrial membrane potential and ATP levels, disrupt mitochondrial dynamic homeostasis, and inhibit antioxidant capacity (31). PS-NP treatment not only elevates pro-inflammatory cytokine production in BV2 cells but also significantly increases ROS generation (**Figures 2F–H**). The elevated ROS levels result in heightened lipid peroxidation, amplified protein oxidative stress, and disruption of cell membrane integrity, ultimately leading to cellular dysfunction and apoptosis (32, 33).

Studies have shown that oxidative stress is a primary mechanism underlying the toxic effects of NPs both *in vivo* and *in vitro*. Wang et al. suggested that the accumulation of PS-NPs induces oxidative stress in loach larvae (34), while Milillo et al. reported that polystyrene nanoplastics mediate oxidative stress in human alveolar epithelial cell lines (35). Our data showed that PS-NP exposure to increased oxidative stress, impaired cognitive function, and activated microglia in mice (**Figures 2E, F**, **3I**, **5H–M**), indicating a multi-level neurotoxic effect. These results confirm that PS-NPs indeed induce oxidative stress and impair antioxidant defense, leading to oxidative stress in the brain and other tissues in mice.

Existing studies have shown that oxidative stress induced by PS-NPs may be associated with the activation of the MAPK signaling pathway (36). Advanced oxidation protein products (AOPPs) induce oxidative stress by activating the MAPK/ERK and MAPK/p38 pathways, leading to epithelial-mesenchymal transition and fibrosis. PS-NP intervention increased ROS levels, which activated the p38 MAPK and ERK pathways in the MAPK signaling cascade through oxidative modification. The activation of these pathways further enhanced ROS production, amplifying the oxidative stress response (37). Our transcriptomic analysis demonstrated that the MAPK pathway was activated in both polyps and BV2 cells following PS-NP treatment. This finding underscores the pivotal role of the MAPK signaling pathway in mediating PS-NP-induced oxidative stress and enhances our understanding of how this pathway operates across various cellular models.

Through the open field test and novel object recognition test, we found that exposure to PS-NPs in mice leads to anxiety-like behavior and cognitive impairment. Nanoplastics can cross the blood-brain barrier, and studies have found that NPs can cause learning and memory deficits, which are major cognitive functions of the brain in zebrafish (38, 39). Sun et al. reported that exposure to PS-NPs induces anxiety-like behaviors in zebrafish (40). Activity among mice in the open field test was significantly reduced following exposure to PS-NPs. This oxidative stress can cause brain tissue damage and potentially impair the normal function of neural cells, leading to anxiety-related behaviors. The novelty score reflects the animal’s preference for a novel object over a familiar one, serving as an indicator of recognition memory. A lower score suggests cognitive impairment. Jin et al. reported that the novelty score of mice in the experimental group was significantly lower than that of the control group, indicating that PS-NPs exposure impaired short-term recognition memory in mice (41). These changes in inflammation and synaptic proteins may collectively contribute to the decline in memory ability in mice.

PS-NPs not only threaten aquatic organisms but also may pose risks to human health through the food chain. Emerging evidence suggests that nanoplastics can cross the blood-brain barrier, potentially contributing to neuroinflammation and neurodegenerative disease. Relevant authorities should prioritize monitoring and establishing stricter standards for plastic production and waste management, particularly in regions heavily affected by pollution. Future efforts should focus on evaluating the potential risks of PS-NPs and developing comprehensive safety management guidelines. Furthermore, public education initiatives should be enhanced to raise awareness of the dangers of PS-NPs and encourage more responsible practices in plastic usage and disposal.

The present study, however, had several limitations. First, the responses of *Aurelia coerulea* polyps and BV2 microglial cells under laboratory conditions may not fully reflect their physiological behavior in natural environments, potentially limiting the generalizability of the findings. Second, only male ICR mice were used in this study, which also presents a notable limitation. This choice was made to minimize behavioral and molecular variability associated with hormonal fluctuations during the female estrous cycle, thereby reducing within-group variance and simplifying the experimental design. Nevertheless, this approach inherently restricts the applicability of the results. Prior studies have demonstrated significant sex-dependent differences in neuroinflammation, oxidative stress responses, and cognitive functions (42). Future studies should therefore incorporate both male and female animals to provide a more comprehensive assessment of the neurotoxic and systemic effects of PS-NPs. Additionally, the exposure duration in this study was relatively short and may not adequately represent the long-term or chronic effects of PS-NPs. To address these limitations, future research should involve multiple species, longer exposure periods, and a broader range of concentrations to better evaluate the ecological and health risks associated with PS-NPs.

In summary, this study reveals that PS-NPs induce oxidative stress via the MAPK signaling pathway, damaging physiological functions, cellular activity, and the nervous system in various models. The findings highlight nanoplastics’ threats to the environment and human health, highlighting a need to develop and improve strategies for protection and safety against PS-NPs.

## 5 Conclusion

The study investigates the oxidative stress effects of PS-NPs on *Aurelia coerulea* polyps and BV2 microglial cells, highlighting the role of the MAPK signaling pathway. PS-NP exposure reduced polyp tentacle length, increased intracellular ROS production, and caused a decline in antioxidant capacity, lipid peroxidation, and cell apoptosis. In microglial cells, oxidative stress induced by PS-NPs triggered an inflammatory response, impacting neural function. In mice, PS-NPs altered oxidative stress biomarkers and caused anxiety and cognitive dysfunction. These findings underscore the potential risks of nanoplastics to marine organisms and the mammalian central nervous system, offering valuable data for assessing their environmental and health impacts. Future research should examine oxidative stress mechanisms under varying concentrations and extended exposure to further assess PS-NPs’ ecological and health risks.

## Data Availability

The data presented in the study are deposited in the NCBI BioProject repository, accession numbers PRJNA1246261 and PRJNA1246183. The accessions are available at the following URLs: https://www.ncbi.nlm.nih.gov/bioproject/PRJNA1246261 and https://www.ncbi.nlm.nih.gov/bioproject/PRJNA1246183 respectively.

## References

[1] Li P Wang X Su M Zou X Duan L Zhang H. Characteristics of plastic pollution in the environment: A review. Bull Environ Contam Toxicol. (2021) 107:577–84. doi: 10.1007/s00128-020-02820-1, PMID: 32166334

[2] Zhou J Chen M Li Y Wang J Chen G Wang J. Microbial bioremediation techniques of microplastics and nanoplastics in the marine environment. TrAC Trends Analytical Chem. (2024) 180:117971. doi: 10.1016/j.trac.2024.117971

[3] Yin K Wang D Zhang Y Lu H Hou L Guo T. Polystyrene microplastics promote liver inflammation by inducing the formation of macrophages extracellular traps. J Hazard Mater. (2023) 452:131236. doi: 10.1016/j.jhazmat.2023.131236, PMID: 36958159

[4] Zhang Y Wang D Yin K Zhao H Lu H Meng X. Endoplasmic reticulum stress-controlled autophagic pathway promotes polystyrene microplastics-induced myocardial dysplasia in birds. Environ pollut. (2022) 311:119963. doi: 10.1016/j.envpol.2022.119963, PMID: 35973452

[5] Avio CG Gorbi S Regoli F. Plastics and microplastics in the oceans: From emerging pollutants to emerged threat. Mar Environ Res. (2017) 128:2–11. doi: 10.1016/j.marenvres.2016.05.012, PMID: 27233985

[6] Ekner-Grzyb A Duka A Grzyb T Lopes I Chmielowska-Bąk J. Plants oxidative response to nanoplastic. Front Plant Sci. (2022) 13:1027608. doi: 10.3389/fpls.2022.1027608, PMID: 36340372 PMC9630848

[7] Smith M Love DC Rochman CM Neff RA. Microplastics in seafood and the implications for human health. Curr Environ Health Rep. (2018) 5:375–86. doi: 10.1007/s40572-018-0206-z, PMID: 30116998 PMC6132564

[8] Wright SL Thompson RC Galloway TS. The physical impacts of microplastics on marine organisms: a review. Environ pollut. (2013) 178:483–92. doi: 10.1016/j.envpol.2013.02.031, PMID: 23545014

[9] Prüst M Meijer J Westerink RHS. The plastic brain: neurotoxicity of micro- and nanoplastics. Part Fibre Toxicol. (2020) 17:24. doi: 10.1186/s12989-020-00358-y, PMID: 32513186 PMC7282048

[10] Ruan Y Zhong Z Liu X Li Z Li J Sun L. Correlation between cellular uptake and cytotoxicity of polystyrene micro/nanoplastics in HeLa cells: A size-dependent matter. PLoS One. (2023) 18:e0289473. doi: 10.1371/journal.pone.0289473, PMID: 37552688 PMC10409258

[11] Lu YY Lu L Ren HY Hua W Zheng N Huang FY. The size-dependence and reversibility of polystyrene nanoplastics-induced lipid accumulation in mice: Possible roles of lysosomes. Environ Int. (2024) 185:108532. doi: 10.1016/j.envint.2024.108532, PMID: 38422876

[12] Amato-Lourenço LF Carvalho-Oliveira R Júnior GR Dos Santos Galvão L Ando RA Mauad T. Presence of airborne microplastics in human lung tissue. J Hazard Mater. (2021) 416:126124. doi: 10.1016/j.jhazmat.2021.126124, PMID: 34492918

[13] Ibrahim YS Tuan Anuar S Azmi AA Wan Mohd Khalik WMA Lehata S Hamzah SR. Detection of microplastics in human colectomy specimens. JGH Open. (2021) 5:116–21. doi: 10.1002/jgh3.12457, PMID: 33490620 PMC7812470

[14] Leslie HA van Velzen MJM Brandsma SH Vethaak AD Garcia-Vallejo JJ Lamoree MH. Discovery and quantification of plastic particle pollution in human blood. Environ Int. (2022) 163:107199. doi: 10.1016/j.envint.2022.107199, PMID: 35367073

[15] Ragusa A Svelato A Santacroce C Catalano P Notarstefano V Carnevali O. Plasticenta: First evidence of microplastics in human placenta. Environ Int. (2021) 146:106274. doi: 10.1016/j.envint.2020.106274, PMID: 33395930

[16] Schwabl P Köppel S Königshofer P Bucsics T Trauner M Reiberger T. Detection of various microplastics in human stool: A prospective case series. Ann Intern Med. (2019) 171:453–7. doi: 10.7326/m19-0618, PMID: 31476765

[17] Kaushik A Singh A Kumar Gupta V Mishra YK. Nano/micro-plastic, an invisible threat getting into the brain. Chemosphere. (2024) 361:142380. doi: 10.1016/j.chemosphere.2024.142380, PMID: 38763401

[18] Sabin KZ Chen S Hill EM Weaver KJ Yonke J Kirkman M. Graded FGF activity patterns distinct cell types within the apical sensory organ of the sea anemone Nematostella vectensis. Dev Biol. (2024) 510:50–65. doi: 10.1016/j.ydbio.2024.02.010, PMID: 38521499

[19] Zeinert LR Brooks AML Couturier C McGaw IJ. Potential use of the Caribbean spider crab Maguimithrax spinosissimus for biofouling removal on marine aquaculture cages. Aquaculture. (2021) 545:737202. doi: 10.1016/j.aquaculture.2021.737202

[20] Wilke CM Wunderlich B Gaillard JF Gray KA. Synergistic bacterial stress results from exposure to Nano-Ag and Nano-TiO(2) mixtures under light in environmental media. Environ Sci Technol. (2018) 52:3185–94. doi: 10.1021/acs.est.7b05629, PMID: 29393629

[21] Fischer R Maier O. Interrelation of oxidative stress and inflammation in neurodegenerative disease: role of TNF. Oxid Med Cell Longevity. (2015) 2015:610813. doi: 10.1155/2015/610813, PMID: 25834699 PMC4365363

[22] Cox KD Covernton GA Davies HL Dower JF Juanes F Dudas SE. Human consumption of microplastics. Environ Sci Technol. (2019) 53:7068–74. doi: 10.1021/acs.est.9b01517, PMID: 31184127

[23] Bajt O. From plastics to microplastics and organisms. FEBS Open Bio. (2021) 11:954–66. doi: 10.1002/2211-5463.13120, PMID: 33595903 PMC8016121

[24] Huang D Tao J Cheng M Deng R Chen S Yin L. Microplastics and nanoplastics in the environment: Macroscopic transport and effects on creatures. J Hazard Mater. (2021) 407:124399. doi: 10.1016/j.jhazmat.2020.124399, PMID: 33191019

[25] Tong Y Lin L Tao Y Huang Y Zhu X. The occurrence, speciation, and ecological effect of plastic pollution in the bay ecosystems. Sci Total Environ. (2023) 857:159601. doi: 10.1016/j.scitotenv.2022.159601, PMID: 36283530

[26] Han Y Lian F Xiao Z Gu S Cao X Wang Z. Potential toxicity of nanoplastics to fish and aquatic invertebrates: Current understanding, mechanistic interpretation, and meta-analysis. J Hazard Mater. (2022) 427:127870. doi: 10.1016/j.jhazmat.2021.127870, PMID: 34848066

[27] Qu M Xu K Li Y Wong G Wang D. Using acs-22 mutant Caenorhabditis elegans to detect the toxicity of nanopolystyrene particles. Sci Total Environ. (2018) 643:119–26. doi: 10.1016/j.scitotenv.2018.06.173, PMID: 29936155

[28] Dash UC Bhol NK Swain SK Samal RR Nayak PK Raina V. Oxidative stress and inflammation in the pathogenesis of neurological disorders: Mechanisms and implications. Acta Pharm Sin B. (2024). 15(1):15–34. doi: 10.1016/j.apsb.2024.10.004, PMID: 40041912 PMC11873663

[29] Janda E Boi L Carta AR. Microglial phagocytosis and its regulation: A therapeutic target in Parkinson’s disease? Front Mol Neurosci. (2018) 11:144. doi: 10.3389/fnmol.2018.00144, PMID: 29755317 PMC5934476

[30] Liu L Xu K Zhang B Ye Y Zhang Q Jiang W. Cellular internalization and release of polystyrene microplastics and nanoplastics. Sci Total Environ. (2021) 779:146523. doi: 10.1016/j.scitotenv.2021.146523, PMID: 34030247

[31] Sun R Liu M Xiong F Xu K Huang J Liu J. Polystyrene micro- and nanoplastics induce gastric toxicity through ROS mediated oxidative stress and P62/Keap1/Nrf2 pathway. Sci Total Environ. (2024) 912:169228. doi: 10.1016/j.scitotenv.2023.169228, PMID: 38101634

[32] Batir-Marin D Boev M Cioanca O Lungu II Marin GA Burlec AF. Exploring oxidative stress mechanisms of nanoparticles using Zebrafish (Danio rerio): toxicological and pharmaceutical insights. Antioxidants (Basel). (2025) 14(4):489. doi: 10.3390/antiox14040489, PMID: 40298867 PMC12024358

[33] Zheng Y Sun J Luo Z Li Y Huang Y. Emerging mechanisms of lipid peroxidation in regulated cell death and its physiological implications. Cell Death Dis. (2024) 15:859. doi: 10.1038/s41419-024-07244-x, PMID: 39587094 PMC11589755

[34] Wang X Jian S Zhang S Wu D Wang J Gao M. Enrichment of polystyrene microplastics induces histological damage, oxidative stress, Keap1-Nrf2 signaling pathway-related gene expression in loach juveniles (Paramisgurnus dabryanus). Ecotoxicology Environ Saf. (2022) 237:113540. doi: 10.1016/j.ecoenv.2022.113540, PMID: 35453027

[35] Milillo C Aruffo E Di Carlo P Patruno A Gatta M Bruno A. Polystyrene nanoplastics mediate oxidative stress, senescence, and apoptosis in a human alveolar epithelial cell line. Front Public Health. (2024) 12:1385387. doi: 10.3389/fpubh.2024.1385387, PMID: 38799687 PMC11116779

[36] Xie X Deng T Duan J Xie J Yuan J Chen M. Exposure to polystyrene microplastics causes reproductive toxicity through oxidative stress and activation of the p38 MAPK signaling pathway. Ecotoxicol Environ Saf. (2020) 190:110133. doi: 10.1016/j.ecoenv.2019.110133, PMID: 31896473

[37] Luo X Wen S Zeng J Liu J Ye W Wu J. AOPPs induces EMT and fibrosis by activating oxidative stress through ERK/p38 MAPK signaling pathway in endometriosis. Reprod Biol. (2024) 24:100950. doi: 10.1016/j.repbio.2024.100950, PMID: 39241657

[38] Kopatz V Wen K Kovács T Keimowitz AS Pichler V Widder J. Micro- and nanoplastics breach the blood-brain barrier (BBB): biomolecular corona’s role revealed. Nanomaterials (Basel). (2023) 13(8):1404. doi: 10.3390/nano13081404, PMID: 37110989 PMC10141840

[39] Zhou W Tong D Tian D Yu Y Huang L Zhang W. Exposure to polystyrene nanoplastics led to learning and memory deficits in Zebrafish by inducing oxidative damage and aggravating brain aging. Adv Healthc Mater. (2023) 12:e2301799. doi: 10.1002/adhm.202301799, PMID: 37611966

[40] Sun Z Wu B Yi J Yu H He J Teng F. Impacts of environmental concentrations of nanoplastics on Zebrafish neurobehavior and reproductive toxicity. Toxics. (2024) 12(8):617. doi: 10.3390/toxics12080617, PMID: 39195719 PMC11359748

[41] Jin H Yang C Jiang C Li L Pan M Li D. Evaluation of neurotoxicity in BALB/c mice following chronic exposure to polystyrene microplastics. Environ Health Perspect. (2022) 130:107002. doi: 10.1289/ehp10255, PMID: 36251724 PMC9555296

[42] Ye M Marzullo B Adler HJ Hu BH. Expression profiling of cochlear genes uncovers sex-based cellular function in mouse cochleae. Hearing Res. (2024) 448:109030. doi: 10.1016/j.heares.2024.109030, PMID: 38776705 PMC11845869

