# Supplementary material for: Polystyrene nanoplastics induce oxidative stress in Aurelia coerulea polyps, microglia, and mice
Source: Front Immunol. 2025 Sep 3;16:1609208. doi: 10.3389/fimmu.2025.1609208 (PMC12440749; doi:10.3389/fimmu.2025.1609208)
Supplement: Supplementary file 1 [file Table1.docx]

Supplementary materials

Supplementary Table 1 Primers for real-time quantitative PCR of Aurelia coerulea polyps

|  | Forward primer (5'to3') | Reverse primer(5'to3') |
| --- | --- | --- |
| GAP | CCGTGTTCCAGTCCCAGATGTTTC | CCTTGCTCTCTGATGCTGCCTTC |
| TAO | ATGTGGCTACTGTGGTGGAGAG | ATCGCTGCTTGTTAGGGTATTGC |
| FGF1 | CTCGTGCGTTGCTGTGCTC | GGATTCATTGCTGCTTGGTTCTTG |
| TRAF2 | GGAGCATTAGAGAACCACCAAAGAC | TCCTCAACCTCCTCAGTATGTAAGAC |
| NGFR | GCCAACAACATCAAGTCAACAACC | TTCGTCGCCACAGGGAAGTC |
| HSPA1_6_8 | CACACATTCTGGTCGCATAAACATAC | ACATCAGTCCTACTTCCTCGCTTC |

Supplementary Table 2 Primers for real-time PCR in mouse BV2 cells

|  | Forward primer (5'to3') | Reverse primer(5'to3') |
| --- | --- | --- |
| GAPDH | AGAAGGTGGTGAAGCAGGCATC | CGAAGGTGGAAGAGTGGGAGTTG |
| CD32 | TGTCGCAGCCATTGTTATTATCCTAG | TGTCTGTACTCACCTACTTCCTCTG |
| CD11β | AGACCTCCAAGACATCAGTGACAG | TCTTCTTCCTCTTCTTCTTCCTCCTC |
| IL-10 | GGACAACATACTGCTAACCGACTC | TGGATCATTTCCGATAAGGCTTGG |
| Arg-1 | AAGACAGCAGAGGAGGTGAAGAG | TAGTCAGTCCCTGGCTTATGGTTAC |
| IL-1β | GAAATGCCACCTTTTGACAGTG | TGGATGCTCTCATCAGGACAG |
| TNF-α | ACGTGGAACTGGCAGAAGAGG | TGAGAAGAGGCTGAGACATAGGC |
| Vegfd | AGTTATAGATGAAGAATGGCAGAGGAC | GGCTTGAAGAATGTGTTGGTTGTC |
| Mapk8ip1 | GGCTGCTGTCTGCGGGTAG | TCCTCCTCCTCGTCGTCCTC |

Continued Supplementary Table 2

| Mapk8ip2 | GGAAGAGGAGGAAGATGGAGATAGG | AGTGTAGTCAGATGGAGAGTGGTG |
| --- | --- | --- |
| Prkcg | CCGACGAACTCTATGCCATCAAG | TGCCAGGACACGCTTCTCTAC |
| Mapt | GATTACACTCTGCTCCAAGACCAAG | GGTTCCTCCGCTCCATCATCG |

**The link to the raw transcriptome data**

The raw transcriptome data has been uploaded to the SRA database. Although it has been uploaded, it has not been released yet. Below is the link provided for the editor's review.

1.Bv2 cells：

https://dataview.ncbi.nlm.nih.gov/object/PRJNA1246261?reviewer=q3kr7b0eiovv3t6jsu9ksaq6h4

2.Aurelia coerulea Polyp

https://dataview.ncbi.nlm.nih.gov/object/PRJNA1246183?reviewer=5kavdud93ka5pqf3o4p2ml5q56
